# Time from Semiosis: E-series Time for Living Systems

**DOI:** 10.1007/s12304-018-9316-0

**Published:** 2018-04-09

**Authors:** Naoki Nomura, Tomoaki Muranaka, Jun Tomita, Koichiro Matsuno

**Affiliations:** 1 Graduate School of Humanities and Social Sciences, Nagoya City University, Nagoya, Japan; 2 Center for Ecological Research, Kyoto University, Kyoto, Japan; 3 Graduate School of Pharmaceutical Sciences, Nagoya City University, Nagoya, Japan; 4 Nagaoka University of Technology, Nagaoka, Japan

**Keywords:** E-series time, The second-person negotiators, Punctuation, Local synchronization, McTaggart, Reaction cycle, Time code

## Abstract

We develop a semiotic scheme of time, in which time precipitates from the repeated succession of punctuating the progressive tense by the perfect tense. The underlying principle is communication among local participants. Time can thus be seen as a meaning-making, semiotic system in which different time codes are delineated, each having its own grammar and timekeeping. The four time codes discussed are the following: the subjective time having tense, the objective time without tense, the static time without timekeeping, and the inter-subjective time of the E-series. Living organisms adopt a time code called the E-series, which emerges through the local synchronization among organisms or parts of organisms. The inter-subjective time is a new theoretical dimension resulting from the time-aligning activities of interacting agents. Such synchronization in natural settings consists of incessant mutual corrections and adjustments to one’s own punctuation, which is then constantly updated. Unlike *the third-person observer* keeping the objective time while sitting outside a clock, *the second-person negotiators* participate in forming the E-series time by punctuating and updating the interface through which different tenses meet at the moment of “now.” Although physics allows physicists to be the only interpreters, the semiotic perspective upends the physical perspective by letting local participants be involved in the interpretation of their mutual negotiations to precipitate that which is called time.

## Introduction

Although we engage in time-conscious life daily, we rarely question the nature of time. However, would it be proper to appropriate our sense of time and scale onto living beings when studying biological worlds? The answer may not be easy, but the following issue should be relevant in biology: how a living being views and measures time. If the nature of living time differs from that of our own, a rethinking of time will shed new light on the temporal aspects of living organisms, expanding the scope of biological investigation.

Clues haves been obtained from several fields, including philosophy and biology. Fraser (2007) proposes the hierarchical theory of time in the universe, from the *proto-temporality* of particle-waves to the *bio-temporality* of living organisms. The species-specific time world has been suggested. According to Cowley and Steffensen (2015), living systems, or organisms-in-the-world, integrate past events with the present to produce anticipated future outcomes; thus, organisms learn to create their own temporal domain.

In terms of semiotic processes, an interpretant is a prerequisite; therefore, it is strange to say that time independently exists apart from the interpretant or organisms. Temporal characteristics in semiotic processes are called *time of being* by Kull (1998) and *radical temporality* by Fernández (2010), and time relations are unmappable onto the coordinate plane that obliterates the interactive dimension of the agential participants there. These studies suggest species-specific temporal domains in addition to our ordinary time.

Time may be thought to frame all events and phenomena in the world as a single objective scale, as practiced in physics, but von Uexküll and Kriszat (1957) clearly states the following:

Time, which frames all happenings, seems to us to be the only objectively stable thing, in contrast to the colorful changes of its contents, and we now see that the subject sways the time of his own world. Instead of saying, as heretofore, that without time, there can be no living subject, we shall now have to say that without a living subject, there can be no time (1957, p. 13).

Where does this upside-down idea come from? Today, we are trained to see a clock as the sole universal index to measure time. In this paper, we take Uexküll’s words literally and explain why his argument is appropriate. The purpose of our paper is as follows: (1) to explain that time is a meaning-making system with its own grammar and methods for timekeeping, and (2) to note that living organisms adopt multiple time codes, including the key code of E-series time (Nomura and Matsuno 2016a, b). E-series time emerges through local synchronization between organisms or parts of organisms.

No one can manifest time in front of us. What can be manifested is a watch or a gadget called a clock, but not time itself. Then, the questions are as follows: who made it, how is it to be read, and by whom? We then notice that time is not a substance but a mode of interaction between active agents. The Earth’s rotation indicates physical movements but not time. The physical movement does not presume time; the interpreter does.

Look at your wristwatch and try to find the past, present, and future. You notice that a clock’s time cannot indicate tense. A clock can measure time, but it has no experiential relevance to you. However, we do grasp what time is all about by experiencing the flow from the past to the present. Perhaps snails experience something similar, too. Even simple creatures must know their past and future in a limited sense to obtain food or to find a mate.

Therefore, here, we imagine at least two distinct times: (1) the subjective time of living organisms with a sense of duration and tense, and (2) externally measured objective time indicating the sequence of an unbroken series of events as epitomized in the succession of a linear sequence of now-points. The latter is tense-less. While subjective time exists in the construction of experience by integrating the past, present, and future at the moment of now, objective time is a construct already completely counted by an external observer (i.e., *the third-person observer*).

## Time as Punctuation

Whether it is subjective (time with tense) or objective (time without tense), time necessitates a reading of some type of scale. The ticking of the hands of a clock is observed to know that time progresses along the objective time scale, whereas our memory and experience framed as sequential events are read according to our past, present, and future on the subjective time scale. In any case, what is noticed and read is a distinction. We call this distinction a boundary. Boundary is an abstraction such as the International Date Line, which is not a dotted line floating in the Pacific Ocean. However, sensing is never possible without distinction made by an organism—whether it is perceptive, auditory, or physical.

Making such a distinction is called *punctuation* in communication theory (Bateson 1972). Punctuation is boundary-making; it brings about differences between warm and cold, right and wrong, day and night, etc. Without distinction made by punctuation, there is no information for the observer. The minimum unit of information is such a distinction, produced and conveyed as the transformation of a difference; “a difference which makes a difference,” as aptly noted by Bateson (1972, p. 459).

Applying the same logic to time, without acts of punctuation, there are no “tick marks” on the time scale. Without “tick marks,” no information is delivered from a clock. Without “tick marks,” we cannot read time. No punctuation, hence, no time, although there is a qualitative difference in punctuation between subjective and objective time. Furthermore, unless punctuation is updated, no sequence of time is expected.

### Inter-Subjective Punctuation

We know that “tick marks” can be set simultaneously by more than one living subject inter-subjectively. “Inter-subjective” refers to the processes taking place among living organisms or among parts of a system, which can also be phrased as “inter-agential” or simply “interactive.” Inter-subjective agents are here called *the second-person negotiators.*

Imagine two percussionists jointly performing, or two kittens being playful with each other. Both achieve their collective action – music or play – by mutually coordinating their physical movements and timing of actions. Neither comes to form itself as an organized whole without coordination. The coordination (i.e., inter-subjective punctuation) is time alignment formed and maintained interactively in the local setting. Inter-subjective punctuation as an alignment of time roughly corresponds to synchronization.

*The second-person negotiators* such as the percussionists and kittens mutually approach synchronization by making communicative moves to each other. The negotiation permits each participant to mutually attend to the punctuations made by its partner. Without assuming the completed result (i.e., objective time), the negotiators follow and act upon each other’s movement to jointly mold of their temporality. The difference between *the third-person observer* and *the second-person negotiators* is substantial. Despite the privilege to declare time objectively, *the third-person observer* has no room or duration of temporality in which mutual interference and disturbance among the local participants are allowed to prevail, even temporarily.

Thus, another type of punctuation is a candidate for a different type of time in addition to subjective and objective time. We call the inter-subjective or inter-agential method of punctuating for synchronization “E-series time” (Nomura 2010; Nomura et al. 2015; Nomura and Matsuno 2016a, b). E-series time is the consequence of local acts of synchronization of an interactive nature and is accessible to *the second-person negotiators* but not to *the third-person observer*.

*The second-person negotiators* and *the third-person observer* differ sharply in their views of the present time. While the present time is an invariant point in the present tense for *the third-person observer* (in the objective time), it is a negotiable temporal zone of “now” for *the second-person negotiators* (in E-series time). The moment of “now” enables local participants to experience and then revise those mutual interferences and disturbances. Revising the interferences and disturbances can thus guarantee updates to acts of punctuation.

## Synchronicity

The phenomenon of synchronization – adjustment of time – is nothing extraordinary. We encounter it in everyday life, for example, in walking together, choreographed dancing, choral singing, or even saying “Amen” together after saying grace before a meal.

The first physicist to hit upon the phenomenon of synchronization is said to be Christian Huygens, who noticed that the resonance of two pendulum clocks hanging on the wall experienced a weak yet definite interaction. The phenomenon of synchronization is colloquially expressed as being “in sync” or “in tune,” but among specialists, the term is also referred to as “entrainment.” Through microscopic analysis of two people engaged in conversation, Condon (1970) identified human conversations as being beautifully synchronized, similar to a pair of good dancers. This phenomenon is called *temporal cohesion* and occurs between participants accessible in second-person negotiations.

### Synchronicity as Time

How then can we relate synchronicity to time? My watch, for example, is roughly synchronized with yours, as well as with the one at the Greenwich Observatory. We also have a long history of counting 24 h based on the Earth’s rotation, synchronizing our time with physical occurrence. Today, our objective time is synchronized with an electronic transition frequency of a cesium atomic clock, which we might call *global synchrony*. On the other hand, our subjective time is synchronized with our own sense of the past, present, and future. The experienced events may be dotted on our *tense scale*, whose congruence is an essential part of our sanity. Thus, both the objective and subjective times can be seen as sign activities of synchronization.

How then is the inter-subjective time (of the E-series) related to synchronization? Think of a pair of good dancers whose steps make their performance smooth and closely coordinated. Each step is a punctuation of his/her own dance, as well as a message to the partner about how he/she is going to move. Each step as a punctuation is a boundary between what has occurred and what is going to occur next. What is required for the two to be in place is mutual adjustment of time in order to make up a unified whole that is constantly in its own making.

Human interactions are filled with synchronization. Our conversation, too, presupposes such coordinated actions through intricate mutual maneuvering among speakers: taking turns in utterance, nodding, or the exchange of other nonverbal cues (Sacks et al. 1974). Conversation requires incessant co-adjustment of timing as if dancing. Such co-adjustment is accessible exclusively in second-person negotiations.

Whether it is dance or conversation, both require local management of time apart from the objective time. Metaphorically speaking, dancing or conversation is considered to be a clock. The underlying question is how to make a larger clock from the interferences between two smaller clocks. The time adopted here is jointly generated by local synchronization, not by the global synchrony of wristwatches.

In biology, many organisms have built-in clocks (e.g., circadian clocks) based on the working of clock genes, producing automatic oscillations of approximately 24 h (circadian rhythm). In mammals, a minute region in the brain called the suprachiasmatic nucleus (SCN) resets internal clocks scattered throughout the body according to light signals from the sun. The resetting of a great number of internal clocks indicates rough synchronization within the single organism.

## Time as a Meaning-Making System

What Uexküll indicated above, and what is encompassed by our argument, implies that time is not substance but is about agential interaction – a meaning-making system shared by the participating agents. Time is constructive or synthetic, so that our fellow philosophers are not the only time makers. This epistemology in mind, we transplanted the theory of time outlined by a British philosopher, J. E. McTaggart (1908, 1927), into the communication domain. Although McTaggart focused on the reality (or non-reality) of time, this study shifts the emphasis by transplanting his scheme to see time as “language” in the broad sense (Takiura 1976).

**Fig. 1:**
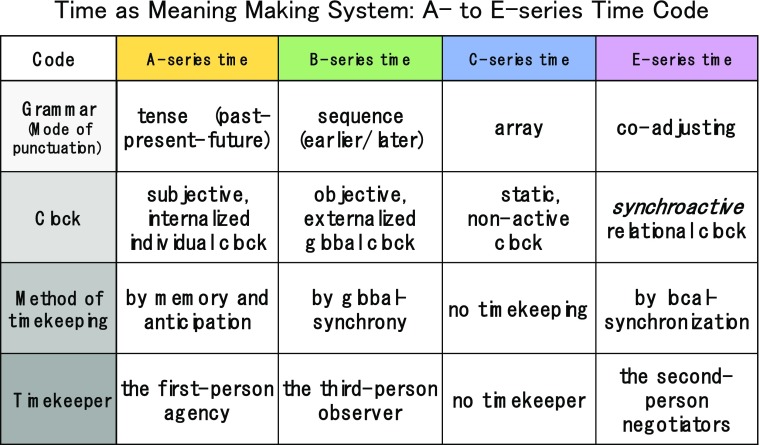
Time as a meaning-making system

McTaggart divided time into three series—the A-series, the B-series, and the C-series—each roughly corresponding to the subjective time, the objective time, and static non-time, respectively. While this is an extremely useful classification, our shift in perspective makes each series a unique method for punctuating and coding (See Fig. 1).

### A-Series Time Code

The key factor of the A-series time code is *tense*: a sense of duration or an unbroken succession of events, which is equivalent to one’s past, present, and future. The A-series corresponds to the subjective time, assuming a single agent having access to its own memory and anticipation. When such flow of time is sensed by *the first-person agency*, time appears to him/her in the A-series. A-series time code is not interactive but intra-agential.

### B-Series Time Code

B-series time code is *tense-less* -- there is no indication of the past, present, and future. It only shows the relations of earlier/later or before/after to be grasped in the third-person panoramic view, which corresponds to objective time. When punctuated sequences of events are seen from the perspective of earlier/later relations in this panoramic view, time appears to *the third-person observer* to be in the B-series. B-series time code is unidirectional and does not assume our participation. Ordinary clocks are such an example.

Note that the genesis of the earlier/later relation is basically subsumed under the tense code: the past, present, and future. When someone walks on the street, the footing of before/after is similar to a living organism differentiating the definiteness in retrospect from the indefiniteness in prospect. Thus, such before/after relations are a part of A-series time, since we can observe tense there in the rudimentary sense. This was the point McTaggart (1908) himself realized; that is, it is self-contradictory to admit definite tenses as a matter of principle on one hand and to say the B-series is derivable from the tense-based A-series on the other. Although McTaggart’s philosophical argument suggests the unreality of time, our study comes to reshuffle the basis of time from its a priori linear progression to semiotic activities.

### C-series Time Code

C-series time code, being a *static array*, indicates neither tense nor earlier/later relations. The punctuations (i.e., tick marks) displayed by clock dials, calendars, timetables, musical scores, and the like themselves are static pictures. A clock’s hands show a simple circular motion. Calendars are composed of equally spaced squares, and timetables are simply a list of numbers. Such time-related compositions – lists, sequences, and patterns – belong to the C-series time code. C-series time code does not assume change – it is “time before time” or simply “pictured time.”

A metronome does not necessarily measure time but can simply be a clicking device. It depends on the interpreter. A metronome itself as a gadget is neither in the B-series nor C-series. If a metronome is seen as an external clock to tell correct time intervals, it appears to be in B-series toward the observer. Of course, it remains in the C-series if viewed simply as a tempo-changing toy.

### E-series Time Code

E-series time refers to inter-agential or interactive time.

#### What Is the Grammar?

E-series time is characterized as inter-subjective punctuation mediated by its constant update, which assumes a local act of synchronization. Its grammar, succinctly put, is as follows: *co-adjusting of time*. That is, one party’s movement for local synchronization turns out to retrospectively disturb the other party’s preceding movement for local synchronization nearby and then lets the other party follow suit. This sequence can repeat itself indefinitely. You may think of conversation or dancing in that the participants coordinate verbal utterances or bodily movements on and on.

The coordination is not concurrent but slightly misaligned (i.e., only finely out of tune on the spot) because such an act of coordination consists of trial and error through which the inevitable temporal misalignments are constantly being corrected. The incessant trial and error creates a certain degree of semiotic freedom (Hoffmeyer 2010), permitting a scope of error or “room” as leeway. It is such “room” that makes systems progress toward the next execution of punctuation, a process that can be integrated into the repeated update of the perfect tense in the progressive tense when viewed from the internalist or local perspective (Matsuno 2015).

When walking on a rope, for example, the walker maintains balance as if dancing by correcting her posture and receiving information coming from the outside. The permitted scope of error makes calibration possible without going outside that scope. Time used on the rope is neither based on her intra-personal subjective narratives (the A-series) nor on the index shown by her globally synchronized wristwatch (the B-series). It must be the one created locally at every moment by adjustment of the walker’s time scale.

#### What Kind of Clock Is it?

During conversation, dancing, rope walking, and kittens playing, coordinated actions are almost simultaneous -- much quicker than sending a message at one time and receiving it slightly later. “*Synchroactive*” may be an appropriate term in this context, more so than simply “interactive.” Each coordinated step is intended to be concurrent with the counterpart, but this very act is slightly out of steps afterwards. It may be joint will on the part of *synchroactive* agents acting as a unit (i.e., as a “clock”) that can maneuver such a quick exercise.

The agent or the participant must read near-future movements in advance and respond seemingly instantaneously. Otherwise, dancing steps, for example, lag behind, and the pair’s bodily movements look awkward and amateurish. The dancers move as a unit as if they know the immediate future on the spot. Since time is in the making, not in the product already made, the participant behaves to leave no disastrous happenstance behind unattended. It is thus *retrocausal* (backward causative) in the sense that the participants act for the present on the basis of the immediate future (Matsuno 2017). This is a type of synchroactive clock available from interactive agents.

#### Who Is the Timekeeper?

Timekeeping in the E-series occurs by multi-agencies in a dialogue-like situation, where progressive states of collaboration are required from one moment to the next. The bonded systemic whole of such inter-relatedness has been called *the second-person negotiators*. The definition, however, is broad enough to cover all the systems that are *closed to information and open to energy* (Ashby 1956). That is, all living systems need energy from outside to sustain themselves, while the internal organization is operated through informational exchange.

Imagine, for example, the rotation of feet pedaling a bicycle, or shoals of fish schooling. The rhythm of turning feet on the pedal and the coordinated swimming without collisions are both the result of time marked in keeping with the surroundings and other individuals. Rotation of the feet interacts with one’s physical conditions, for example, the stiffness of the pedals. Synchronized swimming, which occurs for a variety of reasons, including anti-predator functions, is made possible by a developed response system for adjusting distance and speed to that of neighboring fishes. When these engagements are seen in the communicative perspective, the interacting unit as a whole may correspond to the timekeeper.

Surely, this rhythm-keeping and adjustment of timing occurs whenever there are interacting entities. These may be broadly considered clocks that adjust their tick marks (i.e., timing of punctuation) to the ups and downs of the environment, suggesting that any closed loops of information exchange inside an organization may be considered a clock.

The following section explains the communicative nature of time with three case examples from biological research.

## Biological Illustrations

Living organisms use all time codes except the B-series. Although E-series time may be the main code upon which living systems rely, they also use A-series and C-series time codes in a limited scope. B-series time is reserved for outside observers in the laboratory and field research.

### Cellular Circadian Clocks in Plants

**Fig. 2:**
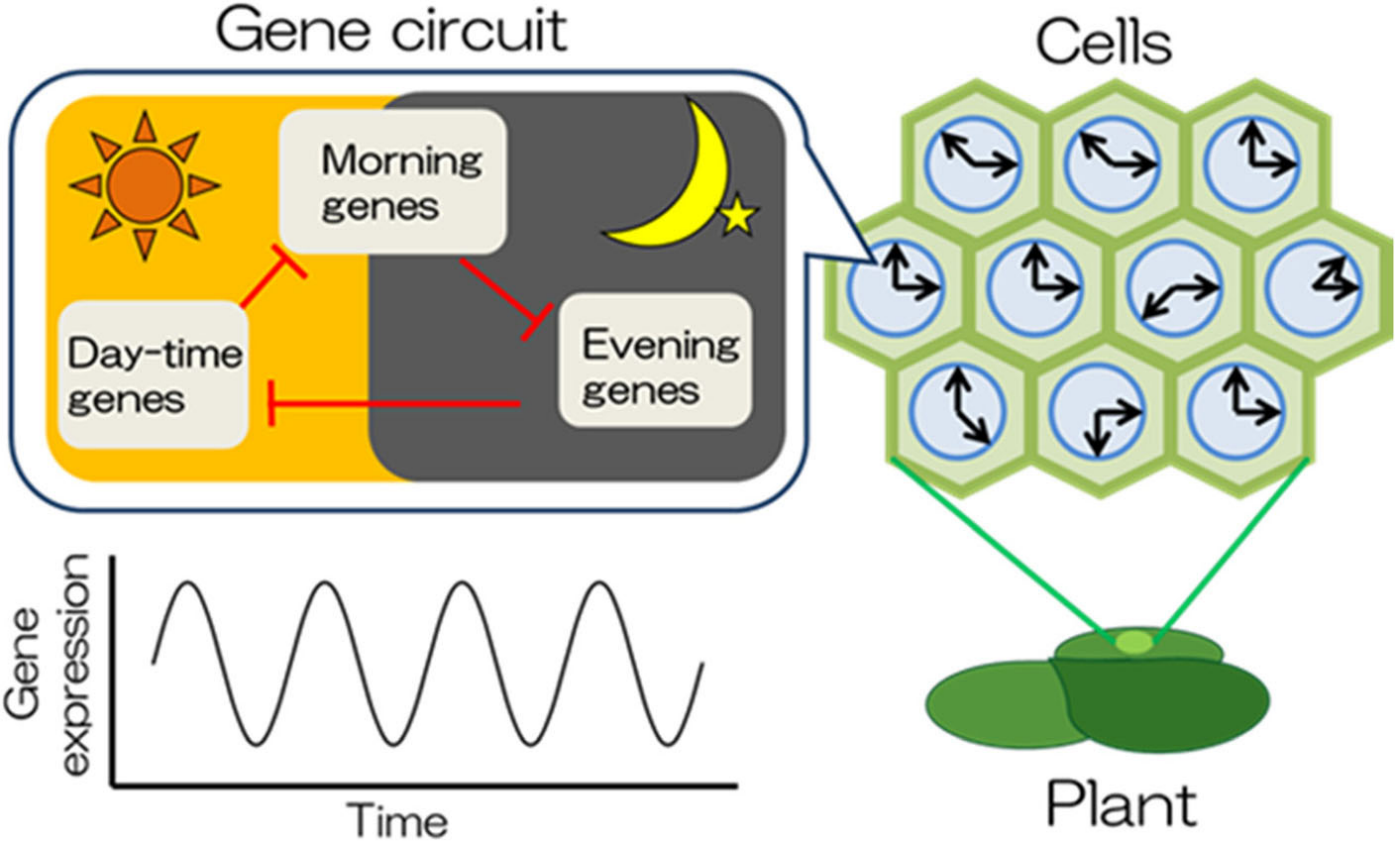
Cells behave as cellular circadian clocks in plants. In a plant body, most cells generate their own daily rhythms using a gene circuit and behave as cellular circadian clocks

The circadian clock is an endogenous timing device based on self-sustained daily rhythms. The rhythms are generated by gene circuits comprised of feedback loops (Pokhilko et al. 2012). Employing a bioluminescent reporter system (AtCCA1::LUC+) using a particle bombardment method, bioluminescent rhythms –endogenous circadian rhythms – are observed in intact duckweed (*Lemna gibba*) cells (Muranaka and Oyama 2016). Each luminesced cell displays the timing of its luminescence peak (i.e., punctuation) reflecting the inner “voices” of clock genes as *the first-person agency* (see Fig. 2). Each cell times its gene expressions, generating rhythms that assume a short-spanned tense.

We thus propose that the feedback loop of the gene circuit can be roughly considered a C-series time code. Fig. 2 shows the organism’s design, which is illustrated by the researcher as a synchronic picture. However, the plant must interpret this to use it diachronically. If not used, the static picture remains as is, as shown in Fig. 2. However, if it is utilized (by a meaning-making organism), the status changes from a static array to an actual chart for behavior, that is, a “scenario” (Hoffmeyer 1996). Regarding meaning-making organisms, C-series time code functions as a “pre-scenario” or a “pre-story” of time, out of which an organism brings its own meanings.

In this way, the sequence of gene expression is “semi-prescribed” in the form of a C-series time code. Each cell reads the chart inscribed in the time code, generating its own rhythms in the A-series, which possess tense. However, the result recorded by scientists, which is the actual sequence of gene expression, gets transformed into the B-series, in which the order of its activation has been fixed.

**Fig. 3:**
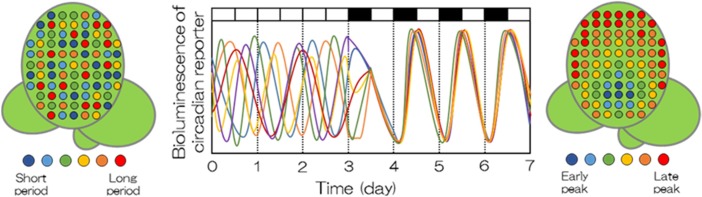
Synchronization of cellular clocks in plants. Cellular circadian clocks in intact plants under constant light conditions were largely heterogeneous, with varied phases and periods. Under light/dark cycles, however, cellular clocks showed spatial patterns at their peak times, indicating that cellular clocks somehow synchronized to each other as well as to light/dark cycles

Under constant light conditions, the rhythm of a single duckweed cell is meaningful only for that particular cell. Each cellular rhythm oscillates with its own period (ranging from 18 h to 28 h) without being affected by neighboring cells. Under light/dark conditions, however, the state of cellular clocks changes remarkably (see Fig. 3). The rhythms of duckweed cells synchronize (or are entrained) to light/dark cycles, and their periods are approximately 24 h (Muranaka and Oyama 2016).

Furthermore, the cells are found to interact with their neighboring cells. The cells mutually observe the rhythms of others in the vicinity, creating spatial patterns for those sharing similar timing of punctuation (i.e., peak times). Thus, the time code used is that of the E-series.

### Kai Protein Oscillators in Cyanobacteria

An example of a circadian clock can also be found in cyanobacteria (*Synechococcus elongatus* PCC 7942). In the case of duckweeds, circadian rhythms are the result of a gene circuit. In cyanobacteria, in addition to the gene expression of *kai* clock genes occurring through transcription-translation, circadian rhythms are produced by Kai proteins themselves, which are the products of *kai* genes.

The three genes generating circadian rhythms are *kaiA, kaiB,* and *kaiC* (Ishiura et al. 1998). Circadian clocks in cyanobacteria were experimentally identified when KaiA, KaiB, and KaiC were mixed together with adenosine triphosphate (ATP) in vitro, and the phosphorylation and dephosphorylation cycle of KaiC proteins showed clear circadian oscillations (Nakajima et al. 2005; Tomita et al. 2005).

KaiC is the central molecule among these three proteins that constitute the circadian clock. When ATP is provided, KaiC forms a hexamer that engages in auto-phosphorylation and auto-dephosphorylation activities (Nakajima et al. 2005; Nishiwaki et al. 2007; Rust et al. 2007). KaiA and KaiB interact with the KaiC hexamer, and each alters the level of phosphorylation – the former helps facilitate auto-phosphorylation, and the latter inhibits the effects of KaiA (Iwasaki et al. 2002; Williams et al. 2002; Kitayama et al. 2003; Xu et al. 2003).

When the circadian oscillation of Kai proteins is experimentally reconstituted, circadian rhythms are observed in each process of *regulation*: (1) auto-phosphorylation and auto-dephosphorylation activities (Nakajima et al. 2005; Nishiwaki et al. 2007; Rust et al. 2007), (2) conformational changes of the KaiC hexamer (Chang et al. 2011; Murayama et al. 2011; Chang et al. 2012), (3) interactions between Kai proteins and complex formation (Kageyama et al. 2006; Akiyama et al. 2008; Qin et al. 2010; Tseng et al. 2014; Snijder et al. 2017; Tseng et al. 2017), and (4) KaiC’s ATP hydrolysis activities (Terauchi et al. 2007; Abe et al. 2015).

The researcher takes samples from the reaction mixture of Kai proteins to examine the above four biochemical processes using several methods and records the changes in each process at scheduled intervals. Time emerges when the continually depicted patterns are punctuated. Objective time of this kind, in which only the earlier-later relationship is discernible, lies in the B-series.

Circadian rhythms of Kai proteins come from four biochemical reactions, which are made possible by the conformation of the KaiC hexamer. Here, we may say that time is mapped out in the structural arrangement of the hexamer since the structural arrangement itself is the circadian time schedule. This time schedule is read by the organism and provides meaning, although the time schedule remains in the C-series before being read. The agency recognizing and reading the C-series text in the context of reconstitution may be intra-molecular KaiC as a subunit and inter-molecular KaiC as a hexamer, as well as KaiA and KaiB.

The four biochemical processes described are mutually dependent and require each other for the creation of rhythms in the circadian Kai protein clock. It is currently hypothesized that KaiC works as a pacemaker by regulating the amount of energy accumulated in KaiC molecules through ATP hydrolysis activity (Terauchi et al. 2007; Ito-Miwa et al. 2016). If so, KaiC as a pacemaker should be able to capture time with tense—the immediate past, present, and immediate future—by observing its own energy level. The energy accumulated inside KaiC through ATP hydrolysis activity should provide markers of the present tense in relation to its past and future, corresponding to A-series time.

With KaiC’s intra-molecular communication within the subunit and its inter-molecular communication in the hexamer, both of which support the four biochemical operations, the resulting periodicity of approximately 24 h is the product of *meta-regulation*, that is, interaction (or communication) among the four biochemical operations.

The four biochemical processes are not independent; therefore, the experimenter cannot measure the rhythms of each process by disregarding the activities of the other three. Circadian rhythms have a certain degree of freedom, some shorter or some longer than 24 h. That is, a certain amount of freedom is permitted, since the rhythms are the outcome of interaction or negotiation between the parts (i.e., *the second-person negotiators*). Because the circadian rhythms are regulated by the interaction of these four biochemical processes, local timing produced through synchronization of the four systemic parts may correspond to E-series time.

### The Citric Acid Cycle

**Fig. 4:**
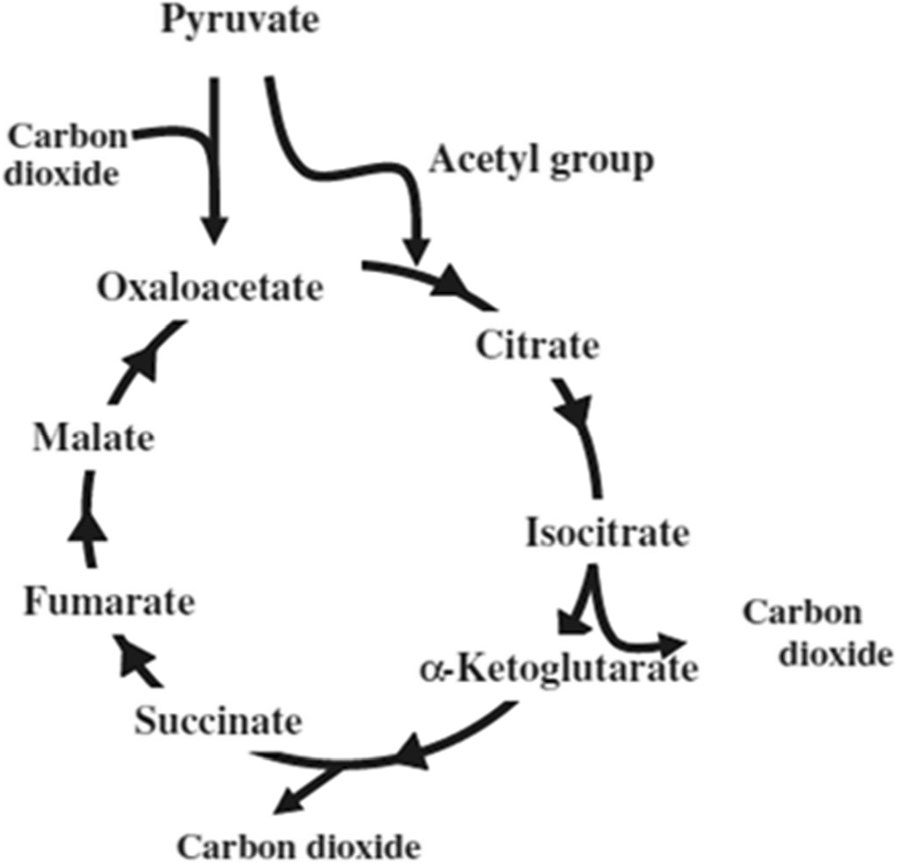
Citric acid cycle

The oxidative citric acid cycle is ubiquitous in the biological world (Buchanan et al. 2000). It is a reaction cycle that synthesizes ATP, in which hydrogen is extracted by the carbon flow circulating through the system, transforming in the direction of citrate to isocitrate, to α-ketoglutarate, to succinate, to fumarate, to malate, to oxaloacetate, and back to citrate via the confluence of acetyl CoA, a co-enzyme (see Fig. 4).

The transformational chain can be considered a design drawing. Thus, Fig. 4 has legitimately been read as C-series time code by the biochemist, the external observer. However, if the participating molecules are taken to assume the semiotic capacity of measuring or being measured by the nearby molecules (Matsuno 2012), it then logically follows that the molecules read a part of the C-series time code, a “pre-scenario,” as indispensable members of the system.

Measurement here corresponds to reading of the code, the “pre-scenario,” through which the molecules select their next step, since measurement is taken to be synonymous with an instance of synthetic chemical reaction (Matsuno 2017). Such measurement is indefinitely repetitive. Like changing partners one after another in certain ethnic dances, detecting the completed molecule partner stipulates the following reaction to come, which may indicate the repeated punctuation of the progressive tense by the perfect tense.

Each molecule takes in the necessary elements for transformation from the outside while leaving the derivative products behind. A molecule balancing both ends of the incoming and outgoing flows indicates time alignment, namely, synchronization between the two material flows occurring at their intersection. Adjusting time at the exchange of material elements indicates the local synchronization of E-series time. From the point of view of each individual molecule as a semiotic agent (Tønnessen 2015); however, the citric acid cycle of the C-series appears filled with *tense markers* of the A-series to be read out.

We should remind ourselves that each citric acid cycle is “the one and only,” that is, each system is unique. The uniqueness is twofold: it resides not only in the cycle itself but also in the relationship each molecule has with others, as well as with the cycle as a whole. This point is vital. Otherwise, the transformation may simply become mechanistic instead of semiotic.

Accordingly, the citric acid cycle as a goal-directed agent orchestrates different processes (i.e., punctuations) around the system, transforming them into time codes. When such a reaction cycle is seen from the first-person perspective, time appears in the A-series, alternating the incumbent tense to reshuffle the constituent elements. The citric acid cycle is thus capable of generating and integrating both its own foresight and hindsight through self-governing activity. That is, each molecule in the cycle *is* both *going to be* part of the reaction cycle to others (the future) and *already* under the influence of the reaction cycle *formed by* others (the past), as figuratively referred to in Uexküll’s Umwelt tunnel (Tønnessen 2014). It is the generation and integration of different tenses at the present moment of “now.” These are the perspectives from the A-series. In contrast, the fixed record of observation abstracted by the external observer as a result remains in the B-series.

On the other hand, timekeeping critical to a reaction cycle is found in the coordination between the sequential transformation of each upstream reactant into the one immediately downstream and the concurrent presence of all the participating reactants. Key to the timekeeping endeavor specific to a reaction cycle of any kind is co-adjustment of the time by multi-participants, despite inevitable conflicts, inconsistencies or incompatibilities between being sequential and being simultaneous.

The reactants view others encountered nearby as *the second-person negotiators*. Who then may be the timekeeper? It would be goal-directed multi-agencies interacting in constant efforts to maintain local synchronization of the E-series, which may correspond at least partially to Gregory Bateson’s concept of *ecology of mind* (1972, pp. 454–471). Biological clocks adapted to circadian rhythms may be no exception in maintaining goal-directed multi-agencies. All clocks seem to take advantage of reaction cycles in one way or another.

## Discussion

The cyclic operations seen in the three biological examples described here are related to clock characteristics. Like stringing beads to make a necklace, the act of connecting often forms a circle. A group engaged in a social activity is colloquially called a “circle.” Whether it is gems, people, or chemical substances, linking something tends to form a circle or a loop. It may be worthwhile to touch upon the general principle of cyclic operation as having clock characteristics. Any closed loop of a reaction cycle, including the gene circuit, the phosphorylation-dephosphorylation cycle, or the oxidative citric acid cycle, has the potential for making and counting time in the E-series when semiotic activity is involved.

**Fig. 5:**
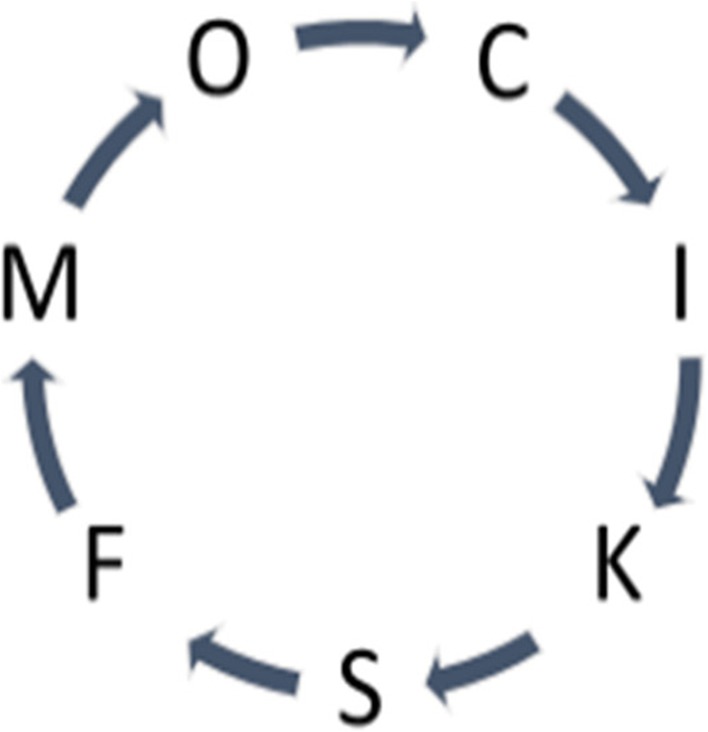
A cycle of transformation

The onset of a reaction cycle must have been a major step in chemical evolution. The reaction cycle is a closed chain with the input of both energy and material substrates. Imagine a cycle of transformation involving reactants C, I, K, S, F, M, and O as constantly taking in necessary resources and letting out the resulting disposed products (See Fig. 5).

Note that the production of any reactant in the cycle appears *causative* in the forward direction as seen in C ➔ O. At the same time, the direct operation C ➔ O in the backward direction appears *retrocausal*. For the reaction cycle to be sufficiently robust, it needs to suppress the retrocausal path (Matsuno 2016). The cycle itself is a picture, which to our eyes is the C-series time code but can be a “text” or “pre-scenario” for the participants or semiotic agents involved.

The reaction cycle in the form of a closed chain assumes chemical affinity between the adjacent molecules. The cohesiveness makes the operation sequential, so that the cycle does not permit the direct transformation C ➔ F, for example, indicating the sequentially scheduled nature of the cycle.

Although cohesiveness, in terms of bond-making, is made possible by sharing an orbital electron (Barbieri 2008), the acts of molecules coming into close encounter with each other to exercise cohesive activity toward others remain semiotic. A molecule can serve as a sign not only for a desired material exchange but also to precipitate bond-making between the two (Matsuno 2012), suggesting messages conforming to two different logical orders: one for a message to *report* its identity to adjacent molecules, and the other for a message to *command* the maintenance of the relationship in the reaction cycle as a whole (Ruesch and Bateson 1951).

When the potential transformation reaction K ➔ S has yet to be materialized, the absence of the reactant S is known by the cycle as a whole, and S is pulled in by it, indicating the activity of chemical affinity. The entire reaction cycle, consisting of the molecules + the cyclic function, is a semiotic agent and comes to know what to expect next. The emergence of reactant S in the reaction downstream is the environment’s anticipatory measurement that the reactant K will be located immediately upstream.

While the reaction cycle as a whole presents a static picture as “text,” which is a C-series time code, the code also possesses information for charting future actions “pre-scenario”.^1^ We may be able to say that “the molecules + the cyclic function” afford each molecule information on how to make the next step in the cycle. Put differently, the relationships displayed in such a semiotic environment can be called *chemical affordance*, extending J. J. Gibson’s original theory (1979). That is, the reaction cycle comes to possess information for each molecule for a “scenario” or a “story.” We also recognize that not only does “the molecules + the cyclic function” together operate as a guide for each molecule but also “the molecule + the cyclic function” as a unit may become the target or object being re-written or re-edited by changes in the behavior of the molecules. The relationship here may consist of two-way reciprocity.

Clock working as a function may be common to all cyclic operations. Whether it is biotic or abiotic, a time frame exists within the reaction cycle for conducting transformative operations with correct timing and in the correct sequence. Imagine Fig. 5 as indicating seven people holding a ringed rope. Just holding the rope and staying still (the static posture) may be equivalent to C-series time. However, once the seven people start moving the rope, say, counter-clockwise, the joint operation must coordinate each move and speed, constituting a cyclic operation. For this, the time code employed is an E-series time code. An outside observer knows how much time is required for ten rounds of circulation in the B-series scale.

If an internal participant were to comment on this state of affairs, he/she would say, “It’s not correct timing, it’s *keeping time* with others.” While timing refers to the state of having been completed, *keeping time* refers to that yet to be completed. That is, timing corresponds to B-series time, which allows all of the past, present, and future to be equally real, but *keeping time* corresponds to E-series time, letting the progressive tense repeatedly update the perfect tense. The internal participants in the reaction cycle know how to manage the latent capacity of the closed chain to build components for clock working.

## Conclusion

Throughout this paper, we have discussed the communicative nature of biological time. Our argument has been that time is a meaning-making system and that living organisms adopt multiple time codes, including the most important one, the E-series, among alternatives. Although we are greatly indebted to McTaggart’s philosophical schema (1908, 1927), our shift in perspective in the present paper is not exactly the extension of it. We interpret time as living codes.

Thus, speaking of its grammar, when we take tense out of the A-series, we arrive at the B-series. When we take both tense and sequence out, we arrive at the C-series. When we revise the structure of the C-series, which consists of repositioning punctuations in the time code, we arrive at the E-series—if the interacting unit (i.e., *the second-person negotiators*) succeeds in effecting mutual time adjustment, and the repositioning does not lead to disorganization of the meaning-making system. In other words, whenever tick marks (i.e., the position of punctuations) are blurred in C-series time code, the system may move either to deteriorated and randomized states (i.e., runaway) or to reformed and reorganized states in a new pattern.

As mentioned earlier, the above relationships also indicate that B-series time can be an abstraction of A-series time simply by reducing the past-present-future tense into an earlier/later sequence of positioning of the countable time. When an anteater captures ants one by one, for example, such subjective time is made up by distinguishing *definiteness in retrospect* and *indefiniteness in prospect* in earlier/later relations. This may be the reason that the earlier/later relation of the B-series is a derivation of the A-series with tense. Let us also remember that the C-series, a “text” or a “pre-scenario,” is supposedly able to “survive” until it meets either a major disturbance from outside or the necessity for revision from within. The latter indicates inner reformations through randomization.

Biological environments permit living organisms to maintain stable organization with a developed “scenario” or a “story” in the citric acid cycle, the Calvin cycle, the gene circuit, and other such examples. Our position is that these “not-yet-activated stories” (pre-scenario) correspond to C-series time of the static time code since the above cycles all subsume time factors. By translating McTaggart’s time schema into living codes, room has been created to accommodate E-series time in the extended theoretical framework.

Despite successive alternations to the members of a reaction cycle appearing in the C-series, E-series time is maintained by “outliving” any members of the C-series time code. Evolutionary processes in biology that manifest in the contents of E-series time must be open-ended as well as endless, while maintaining the time code by incessant updating of “now” (i.e., *keeping time with others*) through the trial and error of collaborating multi-agencies with choices of action. Concrete contents of a new experience or E-series time can be made accessible only after the recording of events in B-series time.

Thus, the relationship between E-series time and B-series time is becoming clear. Time in physics (B-series time) is an a priori condition for *the third-person observer* to approach the motion of physical organization. The observer reaches the present moment from the globally synchronized external time scale. However, in biological worlds, including that of the humans, time is semiotic as the manifold series, as we have shown with examples. The interactive time of the E-series is generated between *the second-person negotiators* using different punctuation or tenses to meet in the negotiable temporal zone of “now.” A decisive issue must be how time is synthesized out of the duration of temporality where mutual inconsistencies, interferences and disturbances are permitted internally.

Finally, we note that the interactional view in general and the meaning-making perspective in particular can offer alternative yet complementary views of the biological world, in contrast to the Cartesian view. Specifically, the four temporal domain series introduced might increase the vocabulary of time language, presenting a more specific picture of the living time code of the E-series. Additionally, by incorporating text characteristics of the C-series within the theory of time, we may be able to connect the synchronic spatial plane (the C-series itself) with the diachronic temporal domain (the C-series being read). Such a scenario might contribute to studies of temporality in biology and biosemiotics in particular.

## Footnotes

1 Uexküll’s key concept, *Umwelt* (Uexküll and Kriszat 1957), may be uniquely related to C-series time. *Umwelt*, the interactive unity of the organism and the world sensed by it, is the subjectively interpreted environment. Living organisms perceive and interpret their own environment, finding its basic dimensions already there, constituting their functional cycle. An organism’s learning of its food, territory, shelter, enemy, etc., constitutes the functional cycle, which is considered the mechanism of *Umwelt*-building (Kull 2001). If *Umwelt* is such a system of signs interpreted by an organism, which is the organism’s way of existing in the environment, it can also be a language used mostly for the internal communication of an organism or lineage (Sharov 2001). Similarly, C-series time, like the objective environment, would remain as it is if the lists, sequence, and patterns are not read and used. Once interpreted, however, the status of the C-series may change from a static array to a scenario or a text: a chart for future behavior. C-series time itself can be viewed as a “pre-scenario” or “text” ready to be read as a time schedule. If *Umwelt,* like the concept of culture for anthropologists, suggests the synchronic picture of a functional cycle, C-series time can be seen as a diachronic picture of a time schedule of *Umwelt*. The details of this relationship need further investigation.
